# Individual Assessment of Brain Tissue Changes in MS and the Effect of Focal Lesions on Short-Term Focal Atrophy Development in MS: A Voxel-Guided Morphometry Study

**DOI:** 10.3390/ijms17040489

**Published:** 2016-04-01

**Authors:** Jan Fox, Matthias Kraemer, Thorsten Schormann, Andreas Dabringhaus, Jochen Hirsch, Philipp Eisele, Kristina Szabo, Christel Weiss, Michael Amann, Katrin Weier, Yvonne Naegelin, Ludwig Kappos, Achim Gass

**Affiliations:** 1 Universitätsmedizin Mannheim, Department of Neurology, Theodor-Kutzer-Ufer 1-3, Mannheim 68167, Germany; J_Fox@web.de (J.F.); eisele@neuro.ma.uni-heidelberg.de (P.E.); szabo@neuro.ma.uni-heidelberg.de (K.S.); 2 Hospital zum Heiligen Geist, Department for Early Rehabilitation, Kempen 47906, Germany; matthias.kraemer@krankenhaus-kempen.de; 3 Institute for Anatomy, Heinrich-Heine-University Düsseldorf, Universitätsstr. 1, Düsseldorf 40001, Germany; thorsten@hirn.uni-duesseldorf.de; 4 Deutsches Institut für Medizinische Dokumentation und Information, Waisenhausgasse 36-38a, Köln 50676, Germany; Dabringhaus@dimdi.de; 5 Fraunhofer MEVIS, Institut für Bildgestützte Medizin, Universitätsallee 29, Bremen 28359, Germany; jochen.hirsch@mevis.fraunhofer.de; 6 Department of Biometry and Statistics, Medical Faculty Mannheim, Ruprecht-Karls University Heidelberg, Mannheim 68167, Germany; Christel.Weiss@medma.uni-heidelberg.de; 7 MIAC, Basel, Universitätsspital Basel, Mittlere Strasse 83, Basel 4056, Switzerland; michael.amann@usb.ch; 8 Neurology, Departments of Medicine, Clinical Research and Biomedical Engineering, University Hospital Basel, Petersgraben 4, Basel 4052, Switzerland; katrin.weier@usb.ch (K.W.); yvonne.naegelin@usb.ch (Y.N.); ludwig.kappos@usb.ch (L.K.)

**Keywords:** regional brain atrophy, multiple sclerosis, voxel, lesion, corpus callosum, lateral geniculate nucleus, shrinking, retrograde degeneration, Wallerian degeneration

## Abstract

We performed voxel-guided morphometry (VGM) investigating the mechanisms of brain atrophy in multiple sclerosis (MS) related to focal lesions. VGM maps detect regional brain changes when comparing 2 time points on high resolution T1-weighted (T1w) magnetic resonace imaging (MRI). Two T1w MR datasets from 92 relapsing-remitting MS patients obtained 12 months apart were analysed with VGM. New lesions and volume changes of focal MS lesions as well as in the surrounding tissue were identified by visual inspection on colour coded VGM maps. Lesions were dichotomized in active and inactive lesions. Active lesions, defined by either new lesions (NL) (volume increase > 5% in VGM), chronic enlarging lesions (CEL) (pre-existent T1w lesions with volume increase > 5%), or chronic shrinking lesions (CSL) (pre-existent T1w lesions with volume reduction > 5%) in VGM, were accompanied by tissue shrinkage in surrounding and/or functionally related regions. Volume loss within the corpus callosum was highly correlated with the number of lesions in its close proximity. Volume loss in the lateral geniculate nucleus was correlated with lesions along the optic radiation. VGM analysis provides strong evidence that all active lesion types (NL, CEL, and CSL) contribute to brain volume reduction in the vicinity of lesions and/or in anatomically and functionally related areas of the brain.

## 1. Introduction

In multiple sclerosis (MS), brain atrophy is a consistent and irreversible feature, as shown in magnetic resonance imaging (MRI) and neuropathological studies [1,2,3,4,5]. Brain atrophy has been adopted as an outcome measure that depicts more permanent tissue damage in studies on disease-modifying drugs [6,7]. The interrelationship between white matter (WM) lesion development and secondary grey matter (GM) atrophy has been demonstrated in several studies [8,9] but little is known about the individual, temporal, and anatomical pattern of brain structure changes after MS lesion occurrence.

In this study we used a method that allows for assessing atrophy development in more detail, regionally and individually. This method, Voxel Guided Morphometry (VGM), uses a non-linear transformation process to register 3D data sets from 2 time points. This allows for following regional volume changes over the whole brain on a voxel-by-voxel basis.

## 2. Results

### 2.1. Lesion Types

**Figure 1:**
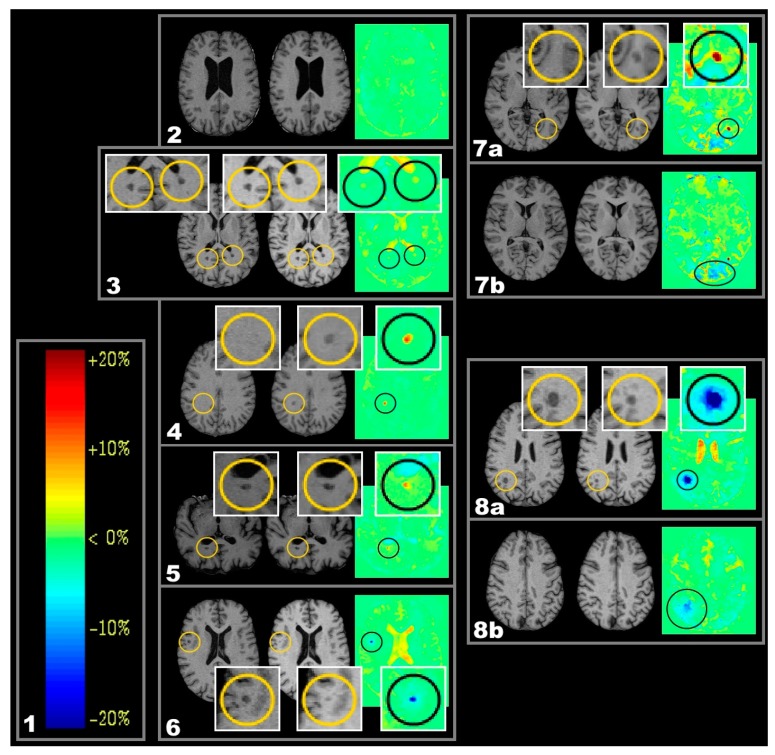
One year follow-up T1-weighted (T1w)) magnetic resonance images (MRI) and respective colour coded voxel-guided morphometry (VGM) maps demonstrating different types of volume change. Important detail is highlighted in circles. (**1**) Colour scale encoding of VGM data. On the right, the volume alteration is given in ten-percent-intervals; on the left, the according colours as depicted in VGM images are given. Stability of brain volume (equivalent to a volume change of 0%) is represented by a light green colour; volume reductions (volume change < 0%) are represented by cold colours, and volume expansions (volume change > 0%) by warm colours; (**2**–**6**) From left to right: MRI-1), MRI-2), and VGM; (**2**) Exemplary case demonstrating lack of volume change; (**3**) Exemplary case demonstrating two inactive lesions, each located close to the posterior horn of the lateral ventricle. They show low volume increase below the cut-off point (light yellow in VGM); (**4**) Exemplary new lesions (NL) (red in VGM and new in MRI-2) located in the right centrum semiovale; (**5**) Exemplary chronic enlarging lesions (CEL) (red in VGM and enlarged in MRI-2) dorsal to posterior horn of the right lateral ventricle; (**6**) Exemplary chronic shrinking lesions (CSL) (blue in VGM and shrunken in MRI-2); (**7a**–**7b**) Exemplary NL that involving fibers of the optic radiation (**7a**). The dependent occipital area shows signals of volume decrease (**7b**); (**8a**–**8b**) Exemplary CSL with light blue halo indicating shrinkage effects along beyond the originally visible lesion (**8a**); this is even visible in a neighbouring slice that did not contain the original lesion (**8b**).

Examples of new lesions (NL), chronic enlarging lesions (CEL), and chronic shrinking lesions (CSL) are depicted in Figure 1, boxes 4, 5, 6, respectively.

Of 89 patients, 69 demonstrated active lesions (NL, CEL, and/or CSL) during the 12 month observation period. In total, 609 active lesions were detected divided into 566 located in the WM and 43 located in the cortical and deep GM (including cerebral cortex, thalamus, and basal ganglia) resulting in 7.1% of lesions affecting the GM. The numbers for NL were 174, 154, and 20 for total, WM, and GM, respectively. In the same order, the numbers for CEL were 262, 256, and 6. For CSL they were 173, 156, and 17. For all 89 patients, this results in averages of 2.0 NL, 3.0 CEL, 2.0 CSL, and 6.8 active lesions (NL, CEL, and/or CSL) per patient (rounded to one decimal place).

Thirty eight patients developed NL, 25/38 developed more than one NL. For CEL and CSL the numbers were 31/49 and 25/48, respectively.

On T1w, the range of lesion sizes was 2–38 mm for NL, 2–70 mm for CEL, and 2–58 mm for CSL with average sizes of 6.0, 11.7 and 9.6 mm for NL, CEL, and CSL, respectively. NL and CEL taken together resulted in an average size of 9.0 mm. Including CSL as well, the average size on T1w was 9.1 mm. The measurement accuracy is at ±2 mm.

### 2.2. Lesion-Related Atrophy of the Corpus Callosum and the Lateral Geniculate Nucleus (LGN)

**Figure 2:**
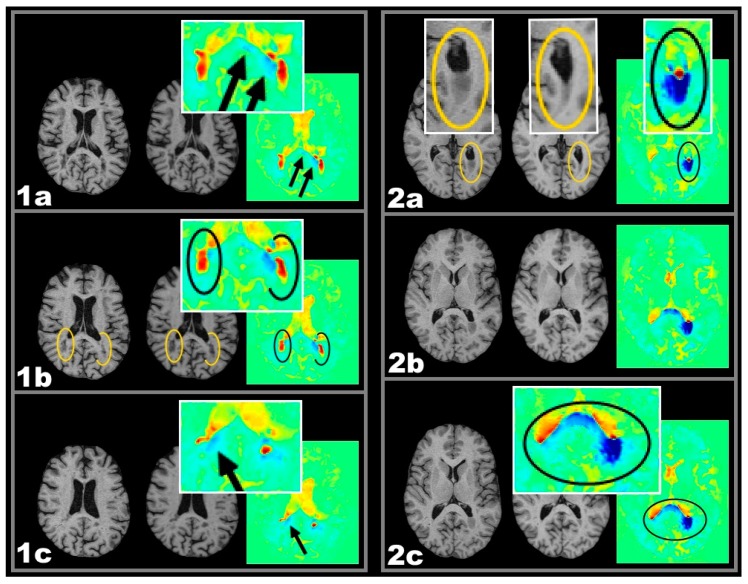
One year follow-up T1w MRI and respective colour coded VGM maps demonstrating different types of volume change. Important detail is highlighted in elipses. Two exemplary cases with active lesions and spatially related callosal volume decrease: Case 1 CEL, Case 2 CSL. From left to right: MRI-1, MRI-2, and VGM. Case 1 CEL: (**1a**–**1c**) Three slices showing bilateral CELs (ellipses in **1b**) close to the posterior horns of the lateral verntricles while callosal shrinkage is observed (arrows in **1a** and **1c**) seen as blue colors; Case 2 CSL: (**2a**–**2c**) Three slices showing a periventricular CSL at the posterior horn of the left lateral ventricle. Adjacent callosal fibers show a volume reduction in the splenium of the corpus callosum. This callosal volume reduction extends to the contralateral hemisphere (**2c**). Important detail is highlighted in circles.

**Figure 3:**
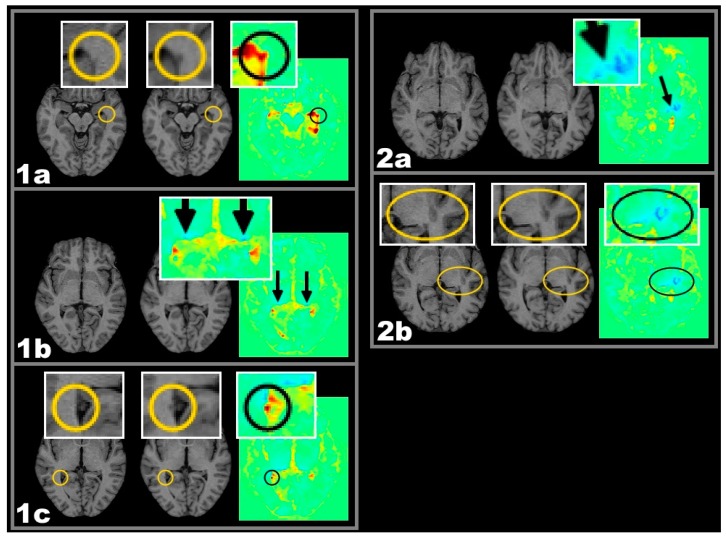
One year follow-up T1w MRI and respective colour coded VGM maps demonstrating different types of volume change. Important detail is highlighted in circles and elipses. Two exemplary cases with active lesions and spatially related lateral geniculate nucleus (LGN) volume decrease: Case 1 CEL, Case 2 CSL. From left to right: MRI-1, MRI-2, and VGM. Case 1 CEL: (**1a**–**1c**) Both LGN of this patient are showing a circumscribed blue VGM signal indicating >5% volume loss (**1b**). Along the optic radiation, this is accompanied by CEL appearance within the left (**1a**) and by NL appearance within the right (**1c**) hemisphere; Case 2 CSL: (**2a**–**2c**) The left LGN area of this patient shows a decrease in size. Lateral to the LGN almost contacting the insular cortex, a CSL is visible with a streak of volume decrease between lesion and LGN (**2a**) possibly representing atrophic fibres of the optic radiation. Important detail is highlighted in boxes and circles. LGN is highlighted by arrows.

As the corpus callosum and the lateral geniculate are typical predilection sites for MS-related tissue damage, these anatomical areas were investigated further. The relationship of active lesions in those areas and their consequences on anatomically connected tissue were further analysed (see also Figure 5). For a definition of the regions of interest see Section 4.5. All subtypes of active lesions in adjacent anatomical regions to callosal fibers or in fibers connected to the lateral geniculate showed effects of tissue shrinkage. Such effects are illustrated in Figure 2 and Figure 3.

#### 2.2.1. Frequencies of Lesions in Patients with Callosal or LGN Volume Reduction

Of the 69 patients with active lesions (NL, CEL, and CSL), 36 patients developed local callosal shrinkage frontally, 34 patients occipitally. Within the frontal (peri-)callosal region 74 CEL, 23 NL, and 22 CSL were found. For the occipital (peri-)callosal region the numbers were 97, 41, and 54, respectively.

In 51 of 138 hemispheres, shrinkage of the LGN was accompanied by lesions in the area of the optic radiation. In total, 26 NL, 57 CEL, and 26 CSL were found along the optic radiation.

**Figure 4:**
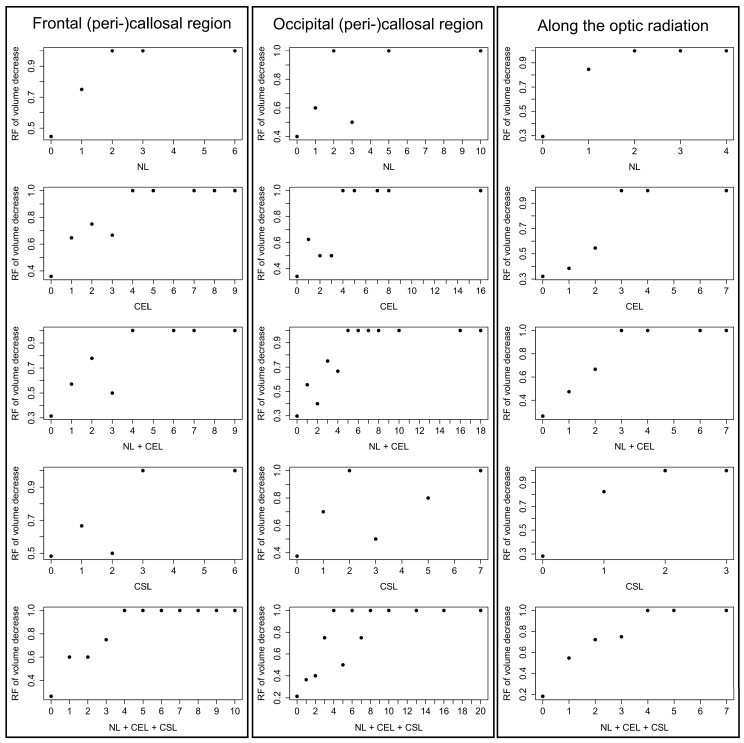
Relative Frequencies (RF) of volume decrease at given lesion counts in individual patients. The **left column** lists the number of lesions (*x*-axis) in the frontal (peri-)callosal region and the relative frequency of callosal volume reduction (*y*-axis). The **center column** considers lesions in the occipital (peri-)callosal region and the relative frequency of callosal volume reduction. The **right colum** considers lesions in the region along the optic radiation and volume decrease of the LGN.

When plotting all observed lesion counts against the percentage of patients showing both this lesion count and local volume decrease, an overall trend was observed (Figure 4).

Each graph in Figure 4 demonstrates one lesion type within one anatomical region. In each graph of Figure 4, all 69 patients were classified according to their lesion count of that specific lesion type within that specific anatomical region. All patients with zero lesions (of this lesion type within this region) are grouped together, as are all patients with one lesion, all patients with two lesions, *etc.* These groups of equal lesion counts are given along the *x*-axis.

Then it was assessed, how many patients within a group showed volume decrease (in the respective anatomical region, *i.e.*, corpus callosum or LGN). This percentage of patients with volume decrease is then plotted along the *y*-axis. Hence, for each lesion-count-group the percentage of patients with volume decrease is shown. If no patients of this group showed atrophy, the *y*-value is zero. If all patients of this group showed volume decrease, the *y*-value is one.

It can be seen that groups of higher lesion counts tend to have higher percentages of patients with volume decrease. Most graphs are even monotonically increasing. Even those that are not monotonically increasing show an overall trend upwards. With remarkably no exception, in all constellations the group with the highest lesion count shows 100% of patients with volume decrease.

A similar pattern is seen for all 3 columns. Both for individual categories of active lesions and for the composite of all active lesions (NL + CEL + CSL) the relative frequency of volume reduction is increasing with increasing lesion numbers.

#### 2.2.2. Logistic Regression Analysis

Results of (peri-)callosal logistic regressions are given in Table 1.

Results of logistic regressions for the LGN are given in Table 2.

#### 2.2.3. Testing for Confounder-Effects of Lesion Types

When plotting the lesion numbers of one lesion type against the lesion numbers of another lesion type, no correlation of lesion types were found–neither for frontal or occipital (peri-)callosal lesions nor for lesions along the optic radiation. All coefficients of determination (*R*^2^) were below 0.1 indicating that there is no need to assume linear correlation between the lesion types.

## 3. Discussion

The mechanisms of brain volume reductions in MS are not fully understood. This study highlights one contributing factor to brain volume changes, namely the lesion related short term evolution of brain volume change in MS. It demonstrates that not only new lesions but also lesions that were already present 12 months earlier can undergo dynamic changes and can contribute to local brain volume loss. We therefore used the denomination active lesion not only for lesions that were newly occurring, but for all lesions that underwent dynamic changes during the 12 month observation period.

However the underlying histo-pathological changes leading to local volume decrease remain uncertain. VGM is unable to differentiate between different histo-pathological states and tissue changes. It is likely that changes in extracellular fluids did occur in lesions and in their vicinity along with other pathological changes (e.g., remyelination, demyelination, astrogliosis). For this reason it is difficult to link a specific mechanism or histo-pathological phenomenon to changes in lesion phenomenology as detected by VGM. Positron emmison tomography ligands in the future will be much better suited to demonstrate specific contributions of inflammatory, neurodegenerative *vs.* edematous changes. This “problem” of lack of specificity applies to almost all MRI techniques, *i.e.*, that the underlying histopathological changes or the the contributions of different mechanisms cannot be determined with certainty. There is however indirect information on this point. Some of the changes in the corpus callosum and the lateral geniculate would favour degenerative axonal changes similar to what has been observed in other MRI MS studies [10,11,12,13].

The results demonstrate quite clearly that preexisting lesions can indeed be seen as a burden in that many of those contribute to the development of local atrophy. Some lesions were stable and showed no signal change over the observation period, however many pre-exisiting lesions demonstrated brain volume changes at a stage when they might be falsely considered as stable chronic pathology. In this regard a recent 11C-PK11195 PET (PK-PET) study demonstrated quite convincingly that T1w so called ”black hole” lesions may have persistent inflammatory activity in the form of microglial activity [14]. They stress that black holes are not just “holes” representing loss of axons and myelin, but may contain inflammatory activity in the form of activated microglia, that they suggested to be relevant in regard to disability progression.

In view of the overall lesion characteristics, the present results fall well within the expected ranges. MS lesion diameters range from a few millimetres to several centimetres with an average of 7 mm [15]. In this study, the average diameter in T1 of NL and CEL combined was 9.0 mm. Additionally including CSL, the average was 9.1 mm. In this study, an average of of 2.0 NL per patient was found by VGM on high resolution T1w MRI in treated patients, which is also in line with previous experiences [15,16]. About 5%–10% of MS lesions may involve the GM, including cerebral cortex, thalamus, and basal ganglia [17]. In this study, 7.1% of all new, enlarging, and shrinking T1w lesions were GM lesions. GM and WM lesions were carefully categorised by visual inspection, which may introduce subjective interpretation in particular in regard to GM lesions in GM–WM boundary areas.

### 3.1. Lesion-Related Atrophy

The results of this work suggest a relationship of focal active lesions with the occurrence of regional volume reduction in anatomically closely related areas of the corpus callosum areas and the LGN. Our analysis suggests that this is the case for all different subtypes of active lesions (NL, CEL, CSL) and not only for one of the 3 subtypes. Since no correlation between lesion types was found (all coefficients of determination were below 0.1), each lesion-atrophy correlation of a specific lesion type may be considered independent from linear effects by lesions of other types.

In order to understand which kind of relationship might underlie the observed tissue changes one may consider the following three possibilities:

1. Since the brain was modelled as an elastic medium, expansion at one location in VGM may be expected to be accompanied by volume reduction in its surroundings and vice versa [18].
2. Independent of the image processing by VGM, there may as well be actual tissue shrinkage due to mechanical compression consecutive to lesion enlargement.

Both explanations interpret the observed lesion-atrophy patterns as due to a general compensatory interrelationship. Hence, in both cases it would be expected that, firstly, all enlarging lesions are accompanied by volume reduction and, secondly, that ovoid lesion enlargement is surrounded by volume reduction in a circumferential manner. Since this was not the case, both interpretations appear inadequate. Furthermore, they fail to explain volume reduction around CSL, which, in the case of volumetric compensation, would be surrounded by volume increase instead. For shrinkage of the LGN, both interpretations may be rejected plainly due to the spatial distance between lesions and the LGN.

3. Therefore, volume reduction associated with lesions located in anatomical and functional relationship might be explained better by assuming a causal relationship. The spatial lesion-atrophy relation appears to be well in line with the possibility of an underlying causal mechanism. e.g., in both cases of Figure 2, lesion location strongly suggests the lesions to have considerable effect on callosal fibres and callosal shrinkage follows the course of those fibres, which are suspected to be damaged.

Since spatial patterns like in Figure 1 and Figure 2 (boxes 7a/b) would be in line with the idea of lesions damaging white matter fibres, there might be a possible chain of causation that would be able to explain the statistically relation between active lesions and regions of volume decrease as atrophic processes in line with Wallerian and/or retrograde degeneration subsequent to lesion-induced fibre impairment [19].

Axonal degeneration, which is considered to be the major cause of irreversible neurological disability in MS [20], may derive from processes such as Wallerian and/or retrograde degeneration [21,22,23,24]. Wallerian degeneration, particularly in neuronal pathways, is assumed to contribute to cerebral tissue loss [17]. Furthermore, cervical cord atrophy has repeatedly been shown not to be associated with focal cervical cord lesions and is therefore considered a sign of long tract degeneration due to damage in remote areas [25,26].

Degeneration of the LGN was reported to follow certain impairments afferent and efferent to the LGN such as the loss of an eye or partial resection of the occipital lobe [27,28]. Mesaros *et al.* found correlations of overall T2 lesion load with thalamic GM loss [29]. However, exact lesion location or internal thalamic topology was not considered which might have served to investigate a functional relationship in more detail. Sepulcre *et al.* demonstrated a correlation between T1w lesions located along the optic radiation and LGN atrophy [8], though without differentiating for the lesions’ volumetric characteristics.

This study corroborates their results and furthermore differentiates between different lesion types according to their recent volumetric development. Since the LGN is the last relay station of the visual pathway before heading for the occipital cortex [30], this lesion-atrophy pattern seems to be explained best according to the idea of retrograde degeneration occasionally accompanied by visible Wallerian (Figure 1 box 7b) and retrograde (Figure 3 box 2a) degeneration along the optic radiation.

#### 3.1.1. Chronic Shrinking Lesions

In case of the chronic shrinking lesions (CSL) one may assume that resolution of extracellular water as part of an inflammatory edema may have contributed to the regional volume reduction. Although we focused our analysis on non-enhancing MS lesions, it is highly likely that resolution of edematous tissue changes have contributed to shrinkage in lesions and possibly also in the surrounding tissue. Although Wallerian degeneration is a likely tissue response to local fiber impairment, we have no exact information on the time of the lesion development and on the timing of the development of Wallerian degeneration. Wallerian degeneration is expected to develop straight away, and it may continue over time resulting in brain atrophy temporally distant from the time point of focal inflammation and demyelination over up to 4 months [7,31].

The finding, in this and other studies, that there are active T1 lesions also demonstrates that models of Wallerian degeneration may need to consider both acute and delayed axonal damage [32]. Further research will need to address this topic.

#### 3.1.2. Limitations

This study has several limitations and is using a new approach to investigate individual lesions in order to learn about their characteristics and effects. Several questions that arise when considering the results were not intended to be addressed at the time point of the design of the study but need to be investigated subsequently in more focussed work.

Due to study design we cannot comment on regional atrophy which may have occurred independent of active lesions. This study focussed on patients showing at least one type of active lesion and their development of regional volume loss in functionally related brain regions. Patients without any active lesions were not assessed further.

We cannot relate our findings in individual lesions to whole brain atrophy amounts quantitatively. However, this study is certainly motivation to investigate this and relate individual or group atrophy measurements to VGM results in order to better understand the quantitative impact of the phenomena of regional volume reduction better.

Our study was not intended to identify clinical factors that would be revealing in regard to the underlying mechanisms of the observed tissue changes. In particular the group of MS patients was rather heterogeneous in regard to their disease duration, disease course and treatment. Further studies will try to identify factors from this spectrum that may be influencing the tissue responses we identified due to active lesions. Most patients were on disease modifying therapies, although not on those nowadays considered the most effective treatments on MRI and clinical outcomes. Clinical confounders, however, cannot be completely ruled out.

## 4. Experimental Section

### 4.1. Patients and Magnetic Resonace Imaging (MRI) Protocol

All patients participated in a study on the phenotypic-genotypic characterization of MS. They were on individually-selected disease-modifying treatments. Inclusion and exclusion criteria are given in Table 3. On the day of the MRI all patients underwent a comprehensive clinical assessment including standardized neurological examination by certified physicians. The 92 patients were diagnosed as follows: 2/92 clinically isolated syndrome (CIS), 69/92 relapsing–remitting MS (RRMS), 16/92 secondary progressive MS (SPMS), 1 patient followed a relapsing progressive course and 4/92 had primary progressive MS (PPMS). Further patient characteristics: 67 women, 25 men, age 32–75 with a mean of 54.5, Expanded Disability Status Scale values 0–7.5 with a mean of 3.0. The mean disease duration was 12 years (range 1–35 years). Patients were treated by individually best selected treatments (Avonex 32/92, Betaferon 12/32, Rebif 44 13/92, Copaxone 4/92, Azathioprin 2/92, Cell Cept 1/92, Tysabri 1/92, no disease modifying treatment 23/92).

Every patient received two 3D volumetric T1w MRI scans twelve months apart (hereinafter referred to as MRI-1 and MRI-2). They were generated with a 1.5 T scanner (Siemens Avanto, Erlangen, Germany) providing a sagittally planned T1w fast, low angle shot sequence (MP-Rage, 160 sagittal slices, slice thickness 1 mm, TR 9.7 ms, TE 4 ms, resolution 1 mm × 1 mm × 1 mm). T2w MRI was not considered for the analysis. Contrast-enhancing lesions were identified on T1w post-contrast MRI but slices containing lesions with contrast enhancement were not included in the VGM defined active lesion category.

Out of originally 92 patients (59 women, 33 men), 3 had to be excluded due to motion artefacts.

Regarding both VGM maps and aligned T1w MRI (see also 2.1), 69 patients developed lesional volume alteration (entirely new lesions, enlarging or shrinking pre-existing lesions) and were therefore investigated further.

### 4.2. Segmentation and Voxel-Guided Morphometry (VGM) Processing

The 3D-MRIs were analysed using a software package that has been previously validated. Voxel-Guided Morphometry uses an automated combined linear and non-linear transformation process to register 3D data sets from 2 or more time points [33,34]. This allows for following brain tissue changes within the whole brain volume on a voxel-by-voxel basis. The transformation of each voxel in the source volume is numerically determined with predefined accuracy resulting in a transformation with approximately 30 millions degrees of freedom.

The main procedures of VGM consist of 5 steps: (1) (preprocessing) In a first automated step, voxels of all osseous structures as well as of dura mater and leptomeninges were removed; (2) Coarse linear alignment by the extended principal axes theory generalized to affine movements; (3) A cross-correlation-based technique using a matrix-norm for fine linear alignment. This is estimated by observer-independent automated definition and correlation of at least 10,000 reference points on the surface of each brain. The combined use of coarse and fine linear alignment results in an exact transformation with respect to size, orientation and position without interactive support; (4) The applied high-dimensional multiresolution full multigrid method determines the nonlinear deformations, resulting in the determination of grey-value guided movement of each voxel from source to target. The resulting high-dimensional deformation field was further processed by (5) determination of volume alterations between MRI-1 and MRI-2. The volume change is indicated on a voxel basis in relation to the neighbouring voxels and was visualized using a colour scale encoding (Figure 1 box 1).

### 4.3. Lesion Types and Cut-off Points for Volume Increase/Reduction

MS lesions are commonly categorized in new lesions (as compared to a prior time point), active lesions, most of the time meaning contrast-enhancing lesions, and chronic lesions, which are pre-existing lesions without contrast enhancement that have a minimum observation period of 6 months.

From a VGM perspective it appears rather more accurate to define active lesions as T1w lesions showing volume increase/reduction in VGM. To minimize false-positive and false-negative results, cut-off points were implemented at ±5% volume change.

Hence, lesions were categorized in active and inactive lesions. Active lesions were either new lesions (NL) on T1w MRI, chronic enlarging lesions (CEL), or chronic shrinking lesions (CSL). Differentiation between NL (defined as volume increase >5% at the location of a new T1w lesion) and chronic lesions from CEL (defined as volume increase >5% at the location of a chronic T1w lesion) was achieved by requiring that in cases of NLs the lesions’ region of volume increase may not overlap with chronic lesions in T1w. In cases of such overlapping, the lesion was classified as a CEL. Differentiation between a single CEL and several CELs within one large chronic T1w lesion was achieved by defining CELs as not in contact with other CELs by their region of volume increase. With the difference of showing volume reduction >5%, CSL were defined analogous to CEL.

The combination of two image modalities, the colour-coded VGM maps and the matching MRI slices from two follow-up examinations served to visually identify the lesions and their location with regard to grey and white matter and differentiate them from partial volume artefacts. There were, however, cortical regions that are prone to partial volume effects and may suffer from residual segmentation errors at the outer brain surface. Furthermore cortical lesions suffer from low contrast-to-noise ratio against the hypointense cortex. Hence, for cortical areas a conservative approach was taken (*i.e*., only lesions exclusively involving GM or clearly centered in the GM were accepted as GM lesions).

### 4.4. Lesion Sizes

In all lesions as detected by the automated VGM algorithm, the lesion diameter was determined by use of axial slices. Lesion sizes were measured to provide reference values in order to estimate the representativeness of the gathered data compared to reports in literature. Since NL only appeared in MRI-2, they, as well as CEL, were measured in MRI-2. CSL on the other hand were determined in MRI-1 since MS lesions may even disappear entirely over time [17,35] and therefore might not appear in MRI-2.

### 4.5. Statistical Analysis—Lesion Related Local Volume Decrease

**Figure 5:**
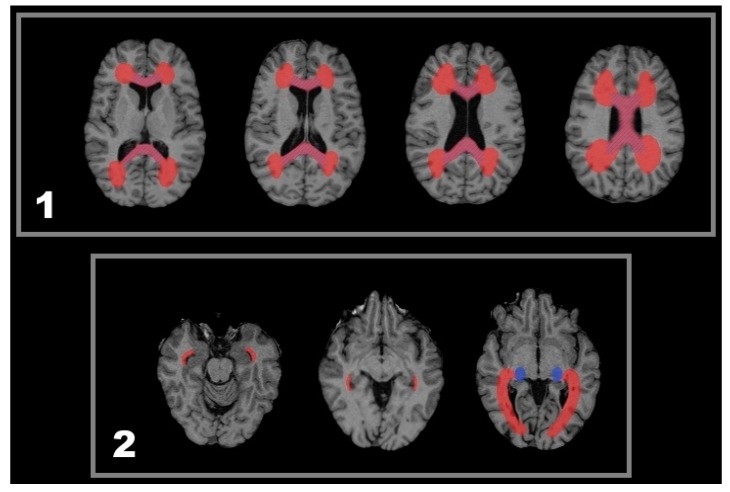
Sketch of anatomical regions of interest regarding the statistical analysis of lesion-related volume decrease. (**1**) The red coloured region delineates a WM region that contains callosal fibres including an adjacent delta-like extension and was searched for lesions. The region investigated for callosal atrophy is drafted in blue hachures; (**2**) In these exemplary slices, a red draft delineates the region covering all three bundles of the optic radiation including the entire lateral and superior wall of the inferior horn of the lateral ventricles [36,37,38,39,40]. For the LGN, a high inter-individual variability is known regarding both size and location [37]. Therefore, a region of the posterior thalamus containing the LGN derived from a population map by Bürgel *et al.* was approximated for each patient so as to assess LGN atrophy consistently (blue draft).

It was assessed whether the occurrence of callosal shrinkage correlated with (peri-)callosal active lesions (*i.e*., active lesions situated close to or within the corpus callosum) since those were considered capable of damaging a considerable amount of callosal fibres (see sketch in Figure 5 box 1). So as, for instance, not to associate lesions of the frontal lobe with atrophy of the splenium, the chosen (peri-) callosal region was narrowed down to a frontal and an occipital half, which were assessed independently. Lesions located in central parts of the callosal truncus were used in calculation for both callosal halfs, since they were assumed to possibly effect fibres of both. However, this merely concerned 2 NL, 4 CEL, and no CSL.

Furthermore, it was assessed whether shrinkage of the LGN correlated with active lesions along the optic radiation.

Descriptions in literature of the course of the optic radiation vary slightly with respect to the coverage of the superior wall of the inferior horn of the lateral ventricles. Though there are many descriptions of the optic radiation covering explicitly the entire superior wall of the inferior horn [37,38,39,40], there are also descriptions of it merely covering posterior parts of the inferior horn [41]. For this study, the entire superior wall of the inferior horn was included in the region of interest (see Figure 5 box 2). This way it could be assured for all three bundles of the optic radiation to safely be accounted for.

Since lesions along the optic radiation of one hemisphere ought not to be interpreted as causes of LGN atrophy of the other hemisphere, this correlation was carried out regarding each hemisphere individually.

### 4.6. Statistical Analysis—Logistic Regression

In this study, several correlations are carried out concerning two characteristics, one of which is dichotomous (*i.e.*, occurrence of parenchymal volume loss), one a not normally distributed quantitative characteristic (*i.e.*, the number of lesions within a functionally connected anatomical region).

According to these requirements, logistic regression was carried out as univariate logistic regression with a dichotomous response variable and a quantitative independent variable.

It was calculated in R (version 3.2.2) including the pROC package for obtaining the ROC curves [42] The logistic models were computed using the glm() function and all *p*-values were determined by the Wald test.

## 5. Conclusions

The analysis of subtle changes in MS lesions over a 12-month interval with VGM reveals active lesions in the form of NL, CEL, and CSL. Active lesions from this perspective are not only new or clearly enlarging lesions, but also shrinking lesions. Active lesions may be accompanied by tissue shrinkage in their vicinity and/or in functionally and anatomically connected fibers and GM. In this regard, brain atrophy as regularly observed as part of the pathological changes in MS appears to be at least partly driven by active lesions. Our findings in the corpus callosum and in the area of the LGN support the contribution of Wallerian and retrograde degeneration to tissue shrinkage as observed with VGM.

## Figures and Tables

**Table 1:** Logistic regressions of lateral geniculate nucleus (LGN) volume reduction and ipsilateral lesion count along the optic radiation. These calculations were carried out separately for the frontal and the occipital half of the corpus callosum. Effect = Euler’s number to the power of the coefficient of the independent variable. AUC = Area under the Receiver Operating Characteristic (ROC) curve. *p*-value = *p*-value calculated with the Wald test. All values rounded to two decimal places, if needed (*p*-values to four decimal places). All *p*-values below 0.05 are accentuated in bold print. Results were not corrected for multiple comparisons.

Lesion Categories	Effect = Odds Ratio	AUC	*p*-Value
new lesions (NL) frontal	4.81	0.63	**0.0338**
chronic enlarging lesions (CEL) frontal	2.21	0.71	**0.0157**
NL + CEL frontal	2.38	0.75	**0.0046**
chronic shrinking lesions (CSL) frontal	1.91	0.57	0.1420
NL + CEL + CSL frontal	2.28	0.77	**0.0027**
NL occipital	2.25	0.64	**0.0345**
CEL occipital	1.99	0.72	**0.0065**
NL + CEL occipital	1.84	0.76	**0.0020**
CSL occipital	1.66	0.67	**0.0307**
NL + CEL + CSL occipital	1.76	0.82	**0.0002**

**Table 2:** Logistic Regressions of LGN volume reduction and ipsilateral lesion count along the optic radiation. Effect = Euler’s number to the power of the coefficient of the independent variable. AUC = Area under the ROC curve. *p*-value = *p*-value calculated with the Wald test. All values rounded to two decimal places, if needed (*p*-values to four decimal places). All *p*-values below 0.05 are accentuated in bold print. Results were not corrected for multiple comparisons.

Lesion Categories	Effect = Odds Ratio	AUC	*p*-Value
NL	14.01	0.65	**0.0006**
CEL	1.90	0.59	**0.0056**
NL + CEL	2.68	0.68	**0.0001**
CSL	12.41	0.66	**0.0001**
NL + CEL + CSL	3.37	0.77	**1.36 × 10^−6^**

**Table 3:** Criteria for inclusion and exclusion of patients.

Inclusion Criteria:	Exclusion Criteria:
Out-patient clinic recruitment (Of 202 recruited patients who participated in a genotype-phenotype multiple sclerosis (MS) study 92 patients were included into this study by random.)	–
Informed consent (in accordance with the approval requirements of the local ethics committee.)	–
Clinical stability (magnetic resonace imaging (MRI) scan of patients with an acute relapse was postponed for at least 30 days after the last dose of steroid treatment.)	–
–	Motion artefacts (This concerned 3/92 patients.)
Lesional volume alterations (This concerned 69/92 patients.)	Volumetric stability over the 12 month observation period (This concerned 20/92 patients.)
